# The potential value of integrated natriuretic peptide and echo-guided heart failure management

**DOI:** 10.1186/1476-7120-12-27

**Published:** 2014-07-18

**Authors:** Maria Chiara Scali, Anca Simioniuc, Frank Lloyd Dini, Mario Marzilli

**Affiliations:** 1 Unità Operativa Malattie Cardiovascolari 1, Dipartimento Cardio-Toracico e Vascolare, Azienda Ospedaliera-Universitaria Pisana, Via Paradisa, 2. 56124 Pisa, Italy

**Keywords:** Biomarkers, B-lines, Echocardiography, Heart Failure, Natriuretic peptide

## Abstract

There is increasing interest in guiding Heart Failure (HF) therapy with Brain Natriuretic Peptide (BNP) or N-terminal prohormone of Brain Natriuretic Peptide (NT-proBNP), with the goal of lowering concentrations of these markers (and maintaining their suppression) as part of the therapeutic approach in HF. However, recent European Society of Cardiology (ESC) and American Heart Association/ American College of Cardiology (AHA/ACC) guidelines did not recommend biomarker-guided therapy in the management of HF patients. This has likely to do with the conceptual, methodological, and practical limitations of the Natriuretic Peptides (NP)-based approach, including biological variability, slow time-course, poor specificity, cost and venipuncture, as well as to the lack of conclusive scientific evidence after 15 years of intensive scientific work and industry investment in the field. An increase in NP can be associated with accumulation of extra-vascular lung water, which is a sign of impending acute heart failure. If this is the case, an higher dose of loop diuretics will improve symptoms. However, if no lung congestion is present, diuretics will show no benefit and even harm. It is only a combined clinical, bio-humoral (for instance with evaluation of renal function) and echocardiographic assessment which may unmask the pathophysiological (and possibly therapeutic) heterogeneity underlying the same clinical and NP picture. Increase in B-lines will trigger increase of loop diuretics (or dialysis); the marked increase in mitral insufficiency (at baseline or during exercise) will lead to increase in vasodilators and to consider mitral valve repair; the presence of substantial inotropic reserve during stress will give a substantially higher chance of benefit to beta-blocker or Cardiac Resynchronization Therapy (CRT). To each patient its own therapy, not with a "blind date" with symptoms and NP and carpet bombing with drugs, but with an open-eye targeted approach on the mechanism predominant in that individual patient. A monocular, specialistic, unidimensional approach to HF can miss its pathogenetic and clinical complexity, which only can be overcome with an integrated, versatile and tailored approach.

## Introduction

Approximately 5.1 million people > 20 years old in the USA live with chronic Heart Failure (HF). An estimated 670,000 new cases are diagnosed annually among USA adults > 45 years old, and HF causes or contributes to almost 300,000 deaths each year. Various demographic trends, including the aging of the population and greater likelihood of survival after acute myocardial infarction, suggest that the prevalence of HF will likely continue to increase; indeed, the American Heart Association (AHA) estimates that by 2030, HF prevalence will increase by 25% over 2013 estimates [1].

Although there have been significant advances in the treatment of HF, morbidity and mortality remain high. Pharmacologic regimens have become increasingly complex, and standard therapy now often consists of multiple drugs (angiotensin-converting enzyme inhibitors, angiotensin receptor blockers, beta-blockers, aldosterone antagonists, diuretics, digoxin, and, in African-American patients, hydralazine and isosorbide dinitrate). The economic impact is significant as well and costs of HF hospitalizations amount to $29 billion/year in the USA alone. Given these epidemiologic and economic pressures, there is increasing interest in using cardiovascular biomarkers for a personalized medicine approach to more effectively guide diagnosis, risk stratification, and therapy [2]. This review aims to provide a reassessment of pathophysiological rationale and existing evidences, highlighting the value and limitations of the currently employed clinical approach based on Natriuretic Peptides (NP), and outline the potential of an alternative, cardiovascular ultrasound-based approach for personalized treatment of HF.

### Biomarkers in HF therapy

In many disease states, drug selection and dosage are strictly dependent from biomarkers [3]. Classic experience with diabetes has taught clinicians to adjust hypoglycemic agents dose to blood glucose levels. The idea of transferring a similar approach to HF may appear attractive. Unfortunately, in HF some basic requirements for such a transposition are missing. HF is a complex systemic syndrome and HF symptoms do not have a consistent relation with severity of Left Ventricular (LV) dysfunction, but express malfunction of adaptive mechanisms, including the natriuretic peptide system, the renin-angiotensin-aldosterone system, and the autonomic nervous system. There is no reason to believe that these systems have a uniform behavior in different forms of HF. Conversely, drug therapy of HF has assumed a uniform response to treatment, irrespective of the pathogenetic mechanism. Based on this oversimplification, HF from global dysfunction of the cardiac muscle, such as in dilated cardiomyopathy, is expected to receive the same treatment as HF associated with regional wall dysfunction, such as in ischemic cardiomyopathy, and even HF in patients with valvular heart disease or hypertrophic cardiomyopathy. Moreover, current guidelines do not consider adjustment of drug selection and dosage to severity of HF symptoms. All symptomatic patients are expected to receive all the agents proven to be beneficial in Randomized Clinical Trials (RCT), at the dosage prescribed in the RCTs, with limited if any room to tailoring of therapy to patient’s need.

Recognizing the heterogeneity of HF and dissecting it into different therapeutic groups would improve the targeting of interventions, which in turn could improve response rates and avoid adverse effects in patients unlikely to benefit. Studies have demonstrated the need to target specific phenotypes based on this heterogeneity [4]. Moreover accurate targeting of therapies could allow the focused use of the drugs most likely to be effective and safe in a given individual, thereby potentially enhancing compliance, improving outcomes, and lowering the cost of medical care. Based on these concepts, it appears that the limitations of current approaches in guiding HF therapy express more the limitations of our understanding of the pathogenesis of these conditions and the simplifications of current therapeutic approach, than intrinsic inadequacy of the employed biomarkers. Heterogeneity in response to therapies warrants further research to identify biomarkers that can not only stratify risk but also identify the underlying disease process that may be targeted by specific therapies.

### Biology of NP in HF

Circulating levels of NP are normally very low in healthy individuals. In response to increased myocardial wall stress due to volume- or pressure-overload states (such as in HF), the Brain Natriuretic Peptide (BNP) gene is activated in cardiomyocytes. This results in the production of an intracellular precursor propeptide (proBNP108); further processing of this propeptide results in release of the biologically inert aminoterminal fragment called N-terminal prohormone of Brain Natriuretic Peptide (NT-proBNP)and the biologically active BNP [5]. In addition, a significant portion of BNP or NT-proBNP detected by current assays includes uncleaved proBNP108, whereas BNP concentrations also include the detection of various subfragments that arise from the degradation of the intact BNP hormone. The biological activity of BNP includes stimulation of natriuresis and vasorelaxation; inhibition of renin, aldosterone, and sympathetic nervous activity; inhibition of fibrosis; and improvement in myocardial relaxation. Although released in a 1:1 ratio, the measured NT-proBNP level is higher than that of BNP, in part because NT-proBNP is passively cleared from the circulation more slowly (half-life of 120 versus 20 minutes). Unlike BNP, NT-proBNP is not cleared by NP receptors or neutral endopeptidases. Rather, NT-proBNP is cleared by various organs, including the skeletal tissue, liver, and kidneys. A common misconception is that NT-proBNP is more dependent on renal function for clearance than is BNP; both are equally cleared by the kidneys.

BNP and NT-proBNP levels are increased in HF, and correlate well with ventricular wall stress and severity of HF. The Breathing Not Properly Multinational Study and the Pro-BNP Investigation of Dyspnea in the Emergency Department showed that NP levels were more accurate for diagnosis or exclusion of Acute Decompensated Heart Failure (ADHF) than clinical judgment, particularly in the context of diagnostic uncertainty. When added to comprehensive clinical assessment, BNP and NT-proBNP are both incrementally useful for diagnosis of ADHF, and both are endorsed in current practice guidelines for HF evaluation (particularly when diagnostic indecision is present). Elevated BNP (above approximately 125 pg/mL) or NT-proBNP (above approximately 1000 pg/mL) values are prognostically meaningful in chronic HF, and a rising pattern is predictive of impending adverse outcome, irrespective of other subjective and objective prognostic metrics. Furthermore, therapies that are favorable for chronic HF (such as beta-blockers, vasodilators, or aldosterone blockers) tend to lower concentrations of BNP or NT-proBNP.

Thus, there is increasing interest in guiding HF therapy with BNP or NT-proBNP, with the goal of lowering concentrations of these markers (and maintaining their suppression) as part of the therapeutic approach in HF. However, recent European Society of Cardiology (ESC) and AHA/American College of Cardiology (ACC) guidelines [6,7] did not recommend biomarker-guided therapy in the management of HF patients. Therefore, in contrast to oncology, biomarker approaches are not yet routinely used in the management of HF. This has likely to do with the conceptual, methodological, and practical limitations of the NP-based approach, as well as to the lack of conclusive scientific evidence after 15 years of intensive scientific work and industry investment in the field.

### Limitations of NP approach

Limitations of NP-based approach include major limitations (including biological variability, slow time-course, poor specificity and lack of conclusive scientific evidences) and minor weaknesses - such as cost and venipuncture.

### Biological variability

The term "biological variability" refers to the extent of changes of a biomarker in a stable physiological state. In practice, biological variability indicates the change in measurement that have a biological significance. Both BNP and NT-proBNP are limited in their clinical use by an excess variability that reaches 40% for BNP and 25% for NT-proBNP [8]. Given a threshold of 1000 pg/mL for the BNP-based diagnosis of cardiac origin of dyspnea, this variability implies that measurements from 600 pg/mL to 1400 pg/ml are biologically equivalent. The negative implications of such variations are obvious in the follow-up of HF patients. For NT-proBNP, the clinical variability is less wide, but still clinically relevant around 25%. In the real world this makes difficult or impossible to estimate if changes BNP plasma levels that remain within this wide range express real changes of patient conditions or only spontaneous biological variability. Trials have used a fixed [9-15] or individualized [16-18] NP target, with no clear difference in results. Six trials testing the value of NT-proBNP used target values ranging from 4000 to 2200 mg/L, with greater benefit observed with higher target values, suggesting that the more aggressive efforts to reduce NP values are not necessarily better.

### Time course of BNP changes in response to therapy

Ideally, a biomarker should reflect as quickly as possible improvement or worsening of patients’ conditions, either spontaneous or therapy-induced. A fast response is even more essential in critical conditions such as decompensated HF. Unfortunately, data from studies of serial BNP measurements suggest that concentrations of NT-proBNP require from 2 to 4 weeks after a therapy change to stabilize [19]. Obviously this makes impossible to rely on these measurements in unstable conditions.

### Poor specificity

Most cardiologists believe that BNP and NT-proBNP directly reflects variations in volume of cardiac chambers. This is certainly true, but by no means a specific finding. In fact, BNP and NT-proBNP levels change also in response to arrhythmias, myocardial ischemia, valvular heart disease, changes of filling pressures, diastolic function, only to mention cardiac factors. Also non-cardiac factors, including age, sex, body mass index and genetic factors are important confounders, as are pulmonary hypertension, pulmonary embolism and chronic kidney disease [20]. NP levels correlate imperfectly with measured filling pressures and may remain elevated in the absence of significant congestion. Some drugs, such as beta-blockers, may increase Ejection Fraction (EF) and improve prognosis, but also increase NP levels through direct pharmacological mechanisms unrelated to effects pump function [21]. Not all causes that elevate NP values are cardiac in origin, and in addition not all cardiac causes of elevation of NP relate to increase in LV pressures and/or volumes. In this era of progressively aging of the HF populations and growing prevalence of comorbidities, the specificity and hence the diagnostic value of NP is strongly challenged.

### Lack of conclusive scientific evidence

In the era of evidence based medicine, no guideline can be proposed if not supported by sound data. More than 9 trials have been completed and published since when NP levels have been proposed to guide HF therapy. The natriuretic peptide guided- strategy is associated with reduction in all-couse mortality and HF- related hospitalization as well as with the risk of readmission for HF worsening, as shown by recent meta-analyses. However further evidence is still needed to support a more general use of NP, since the results of this trial are inconclusive as regards cardiac death [22,23]*.*

The results of these trials are inconsistent and inconclusive. The clear demonstration of this statement is that an additional trial is being conducted and eagerly waited by both supporters and skeptics (Guiding evidence based therapy using biomarkers intensified treatment, GUIDE-IT). The mixed results are after all not so surprising in the light of previous limitations. NP increase is determined by a spectrum of different pathophysiological conditions (from lung congestion to systolic dysfunction to increase of mitral insufficiency during exercise) and triggers a monotonous therapeutic response, of questionable benefit in the individual patient, and typically associated with a relative increase in the use of angiotensin-enzyme inhibitors, beta-blockers, angiotensin receptor blockers, and spironolactone over standard, symptom-guided care. If the physician relies on NP levels to assess volume status, the choice of dose escalation of loop diuretics in patients without pulmonary congestion may increase the rate of hypotension, renal dysfunction and adverse outcome.

### Cost

HF poses a heavy economic burden on western economies, being one of the largest contributors to health cost. NP measurements, though not very expensive add to the overall cost of HF therapy. The additional cost for each BNP measurement varies largely from hospital to hospital, and averages 30 €. NT-proBNP measurement is generally more expensive. Given the need for serial measurements and the number of the HF populations, it is easy to estimate how much a systematic NP based approach would impact on health cost. That is why a generalized use of NP based therapy is not justified until conclusive evidence is provided of its cost-effectiveness.

### Need for venipuncture

This may appear as a minor nuisance for doctor and patients, but sometimes the blood sampling may be a challenging task in elderly, fragile, obese patients.

Based on the concepts expressed in preceding paragraphs, therapy of HF might be reconsidered. An effort should be made to identify the agents and the dosages to be prescribed to patients according to the etiology of the HF and to the severity of symptoms, abandoning the current strategy of giving all agents, at the highest tested dose, to all patients. It is conceivable that in this context, patients can be identified in whom NP dosage may provide a good guidance to therapy, and patients that do not have benefit from such approach. This hypothesis would become even more appealing if biomarkers with less biological variability, greater specificity and faster time response can become available.

### The potential role of echocardiographic biomarkers in heart failure

Echocardiography-derived parameters can fulfill the definition of biomarkers since in 2001, a working group of the National Institutes of Health standardized the definition of a biomarker as a "characteristic that is objectively measured and established as an indicator of normal biological pathologic processes, pathogenetic processes, or pharmacologic responses to a therapeutic intervention". A biomarker may be measured on a biosample (such as a blood test, for instance, the D-dimer as a biomaker of vulnerable blood) or it may be an imaging test (for instance, echocardiogram for vulnerable myocardium) [24]*.*

The ideal candidate biomarker should fulfill some basic pre-requisites: 1) easily defined, safe, and reproducible in each patient; 2) appropriate therapeutic counter measures should be available to reset the abnormal biomarker values towards normal range; 3) the biomarker-triggered therapy change should improve outcome; 4) the biomarker-driven approach should provide an improved benefit and cost-benefit over standard approach.

A classical example of a powerful risk marker unsuitable as a therapeutic target is serum sodium concentration. The lower the sodium concentration, the higher the degree of neuro-hormonal activation and the poorer the prognosis, but treatment aimed at normalizing sodium levels will not improve clinical outcomes [25].

Echo biomarkers in HF show several prerequisites for being useful. According to the recent ESC guidelines on HF, echocardiography is the imaging method of choice for reasons of accuracy, availability (including portability), safety and cost. The more frequently used echo parameters are reported in Table 1. They are certainly simple to measure, even with pocket-size instruments, and reasonably reproducible in expert hands. They are associated with strong prognostic power, and several have shown independent and incremental prognostic value over standard clinical and bio-humoral predictors. They explore different and complementary aspects of HF pathophysiology including: LV function (usually with EF with biplane Simpson method, or with more accurate but also more costly and technically demanding Real Time 3D); right ventricular function (with tricuspid annular plane systolic excursion); diastolic function (with left atrial volume index, E/e’ ratio, E wave deceleration time); extravascular lung water (exploring pulmonary congestion with lung B-lines); mitral-insufficiency; pulmonary hypertension (with pulmonary artery systolic pressure). Another extremely attractive field is the assessment of the behavior of these markers during exercise. Stress echo applications beyond coronary artery disease are emerging as an attractive field, and there is no question that a moderate mitral insufficiency becoming severe during stress and accompanied by B-lines and pulmonary hypertension can make a mitral valve repair an attractive therapeutic option. However, also in these cases the evidences on points 3 and 4 on the chain of biomarker validation are conspicuously lacking to date.

**Table 1:** Common echocardiographic and lung sonography abnormalities in patients with heart failure

**Measurement**	**Abnormality**	**Clinical implications**
**Parameters related to systolic function**		
LV ejection fraction	Reduced (<50%)	LV global systolic dysfunction
RV Tapse	Reduced (<16 mm)	RV global systolic dysfunction
**Parameters related to diastolic function**		
E/e’ ratio	Increased (>15)	High LV filling pressure
**Parameters related to pulmonary congestion**		
B-lines	> 5 in anterior chest scan	Extravascular lung water
**Parameters related to valvular function**		
Mitral valve dysfunction	Severe mitral regurgitation	Cause/consequences of HF
**Parameters related to contractile reserve**		
Global LV function during stress	No contractile reserve	Unresponsive scar tissue

LV, left ventricle; RV, right ventricle; TAPSE, tricuspid annular plane systolic excursion. Adapted from ESC 2012, ref 17.

### Limitations of echocardiographic approach

In spite of the unsurpassed appeal in terms of low cost, widespread access, versatility of the information provided, there are limitations of echo-based approach, including dependence on patient’s acoustic window, operator’s expertise and lack of sufficient evidence based-data in echo-driven management of HF patients [6]*.*

### Dependence on patient’s window

Although last generation echocardiographic instruments allow satisfactory to excellent imaging in > 90% of patients referred to echocardiography lab, poor acoustic windows exist in a significant portion of subjects, for instance with morbid obesity or lung disease. The use of intravenous contrast agents for endocardial border enhancement and better left ventricular cavity recognition is indicated whenever ≥2 contiguous segments are not adequately visualized [26] and considerably increases the success rate of echocardiographic imaging, but with an extra cost. In practical terms this implies that out of 10 patients candidate for enrollment in an HF trial using an echo-biomarkers only 8 to 9 can be considered eligible for presence of good quality of echocardiograms allowing to have not only interpretable but also measurable tracings.

### Dependence on operator’s expertise

The echocardiographic examination is highly dependent on the quality of image acquisition and analysis. Therefore, strict criteria of image acquisition, storage and analysis are required when echocardiographic data are used in a clinical trial. The American Society of Echocardiography issued some recommendations on these aspects, suggesting that only certified and trained sonographers are involved in data acquisition, and in multicenter trials core lab reading by experinced operators blinded to patient identity and study condition is recommended [27]. In addition, whenever possible a quantitative approach to image analysis should be used. In fact, several quantitative approaches have been recently proposed to evaluate ultrosound biomarkers with an operator-independent, quantitative approach for instance to assess left ventricular function with 2D speckle tracking technology [28] This will increase the precision of the method and reduce the sample size required to achieve the required statistical power of a trial [27].

### Lack of sufficient outcome data

Although a large number of observational studies support the use and prognostic values of several echocardiographic markers of HF, we still miss prospective randomized trials based on echo parameters as a guide to treatment. When this has been done in other fields, results have not been always in line with the expectation and conventional wisdom. For instance PROSPECT study did not confirm that mechanical dyssynchrony with echo is associated to better outcome with CRT therapy [29]; COURAGE did not show that ischemia-driven revascularization improves prognosis over optimal medical therapy in patients with stable angina [30]; and STICH trial did not show that myocardial viability is associated to better prognosis in revascularized subjects with left ventricular dysfunction over medically treated patients [31]*.*

It is obvious at this point that we need stronger evidence from prospective randomized outcome data before we can use echocardiography to guide our therapy choices in heart failure.

## Conclusion

**Figure 1:**
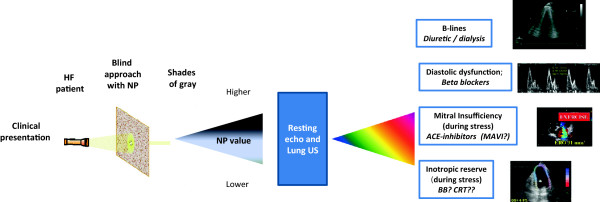
**From natriuretic peptide driven (without imaging support) to "echo-print" individualized and tailored treatment in heart failure: different echocardiographic fingerprints ("echo-print") have different therapeutic implications.** For instance, evidence of pulmonary congestion (right upper panel) through B-lines supports the use of diuretic treatment, whereas the lack of contractile reserve during dobutamine stress echo (right lower panel) discourages the indication to CRT.

An integrated ultrasound assessment with echocardiographic and lung ultrasound, in resting conditions and even better during stress [32], can provide a spectrum of pathophysiological and hemodynamic information, which can conceivably be mirrored in selective, personalized, tailored therapeutic decisions (Figure 1).

The concept of the application of biomarkers as a guide to therapy is certainly an interesting one, which has stood the test of time. Not all HF patients with the same clinical picture were created equal, and NP assessment identifies higher and lower risk subgroups, which may help in guiding a different level of treatment intensity. We have learned in the past 15 years that the good clinician needs little help in starting an appropriate therapy of HF patient, but even the best clinician needs help in tailoring the best therapy in the HF patient to prevent acute decompensation or catastrophic complications. Many attempts in these directions including the simple NP-guided therapy met only limited success with uncertain impact on mortality. On the light of the evidences presented, this cannot be considered surprising since the increase in NP is a monotonous response to a variety of different patho-physiological input conditions and, furthermore, it evokes a rather monotone output response with intensification of drug treatment. This simple and straightforward diagnostic and therapeutic algorithm can be beneficial in some patients, neutral in others, and detrimental in a subset. For instance, an increase in NP can be associated with accumulation of extra-vascular lung water, which is a sign of impending acute heart failure. If this is the case, an higher dose of loop diuretics will improve symptoms. However, if no lung congestion is present, diuretics will show no benefit and even harm. It is only a combined clinical, bio-humoral (for instance with evaluation of renal function) and echocardiographic assessment which may unmask the pathophysiological (and possibly therapeutic) heterogeneity underlying the same clinical and NP picture. Increase in B-lines will trigger increase of loop diuretics (or dialysis); the marked increase in mitral insufficiency (at baseline or during exercise) will lead to increase in vasodilators and to consider mitral valve repair; the presence of substantial inotropic reserve during stress will give a substantially higher chance of benefit to beta-blocker or CRT therapy [33-35]. To each patient its own therapy, not with a "blind date" with symptoms and NP and carpet bombing with drugs, but with an open-eye targeted approach on the mechanism predominant in that individual patient. A monocular, specialistic, unidimensional approach to HF can miss its pathogenetic and clinical complexity, which only can be overcome with an integrated, versatile and tailored approach. This is a rational clinical strategy today, but it is also a roadmap for future research, which should promote outcome studies with echo-driven therapy compared with the standard approach. At least one large-scale, prospective, randomized trial is already in progress in high-risk chronic kidney disease patients with heart failure and renal insufficiency and on dialysis—the LUST Trial (Lung water by UltraSound guided Treatment to prevent death and cardiovascular complications in high-risk end-stage renal disease patients with cardiomyopathy) [36]. The results of this study will be crucial eventually to incorporate B-lines into our clinically oriented diagnostic algorithms. Another single-center trial comparing the outcome of B-lines driven therapy versus standard therapy in ambulatory outpatients is ongoing [37]. At this point, the stage is set for prospective trials comparing outcome in standard (or NP-based) [38] versus echo-driven tailored therapy of HF, moving from a blind, bio-humoral, one-fits-all approach to an open-eye, imaging-guided, therapeutically versatile approach to the patient with HF. In the management of HP patient, even the best cardiologist needs help – but the help provided by NP is costly, pathophysiologically ambiguous, with clear additional economic burden and unclear benefit of outcome. The "peptide-diuretic reflex" can even be detrimental, as the oculo-stenotic reflex of an anatomy-driven coronary revascularization. We need a better way to titrate and personalize therapy in our HF patient (Table 2). It is up to the cardiology and echocardiography community to build the missing evidence required to shift the practice of HF from art of clinicians to evidence- based tailored and individualized treatment of HF guided by low cost portable radiation-free imaging techniques [39,40].

**Table 2:** Biomarkers in heart failure

	**What we have**	**What we need**
**Clinical presentation**	Physical exam + chemistry	Safe and low cost Imaging
**Biomarkers**	Natriuretic peptides	Echo-print
**Drug treatment**	One fits all	Personalized and tailored
**Evidence for echo-print**	Proof of concept	Proof of efficacy

## Abbreviations

ADHF: Acute decompensated heart failure; AHA/ACC: American heart association/American College of Cardiology; BNP: Brain natriuretic peptide; CRT: Cardiac resynchronization therapy; ESC: European Society of Cardiology; EF: Ejection fraction; HF: Heart failure; LV: Left ventricular; NP: Natriuretic peptides; NT-proBNP: N-terminal prohormone of brain natriuretic peptide; RCT: Randomized Clinical Trials.

## Competing interests

The author(s) declare that they have no competing interests.

## Authors’ contributions

MCS conceived the study and drafted the manuscript which was critically reviewed by AS, FLL and MM. All authors read and approved the final manuscript.
